# Porogen-Mediated Barrier Control in Multilayered Drug-Eluting Antibacterial Films: Comparative Evaluation of PEG, PVP, and PEOx

**DOI:** 10.3390/pharmaceutics18060736

**Published:** 2026-06-13

**Authors:** Sergey G. Poroshin, Arkady S. Abdurashitov, Gleb B. Sukhorukov, Pavel I. Proshin

**Affiliations:** 1 Center for Bio- and Medical Technologies, 30 Bolshoy Boulevard, Bld. 1, Moscow 121205, Russia; 2 Life Improvement by Future Technologies Center (LIFT Center LLC), 5 Nobelya Street, Moscow 121205, Russia

**Keywords:** drug-eluting films, porogen, polyvinylpyrrolidone, poly(2-ethyl-2-oxazoline), PEG, local drug delivery, antibacterial films, controlled release

## Abstract

**Background:** Polymeric drug-eluting films are promising platforms for local antibacterial delivery, but their release profiles depend strongly on the permeability and morphology of the barrier layer. Here, the previously proposed concept of additively manufactured PLACE (Printed Layered Adjustable Cargo Encapsulation) coatings was extended from "single orifice"-defined release toward porosity-assisted barrier control. Two conventional water-soluble porogens, polyethylene glycol (PEG) and polyvinylpyrrolidone (PVP), were compared with poly(2-ethyl-2-oxazoline) (PEOx), a hydrophilic polymer proposed as an alternative to PEG in biomedical formulations, but whose use as a leachable porogen has received little attention. **Methods:** Each porogen was introduced into the upper PLGA barrier of multilayer PLACE films. The resulting films were characterized for film formation, post-hydration morphology by SEM, release of methylene blue and vancomycin, and antibacterial activity against methicillin-resistant Staphylococcus aureus (MRSA). **Results/Conclusions:** PEG was poorly compatible with PLGA and mainly produced surface-localized defects rather than a barrier with controlled permeability suitable for prolonged delivery. PVP K17 provided sustained release at 10 wt.%, whereas 20 wt.% PVP caused burst-dominated release and stronger morphological disruption. PEOx formed developed porosity at lower loading and produced release regimes ranging from several days to approximately two weeks. Vancomycin-loaded films containing 5 wt.% PEOx enabled near-complete release over two weeks while preserving film integrity and showed pronounced early anti-MRSA activity. These results identify porogen selection as a key formulation step and support PEOx as a useful porogen for early high-output antibacterial PLACE coatings.

## 1. Introduction

Polymer-based drug-eluting films are well suited to localized antibacterial therapy. They sustain therapeutic concentrations directly at the target site and limit systemic exposure to the drug. This makes them useful for preventing or controlling localized infections at tissue-facing or implant-associated surfaces, where sustained local release can offer advantages over repeated dosing or systemic administration [1,2,3]. In such platforms, release is determined not only by the drug and matrix polymer, but also by the transport properties of the barrier separating the drug-loaded compartment from the surrounding medium [4,5,6].

Reservoir-style delivery devices are traditionally regulated either by a defined delivery orifice or by a porous barrier that controls water ingress and drug transport [7,8,9]. PLACE (Printed Layered Adjustable Cargo Encapsulation) films have previously been introduced as additively manufactured, planar, reservoir-like coatings fabricated by layer-by-layer deposition with a programmable CNC dispenser [10]. In this architecture, the drug-containing reservoir and the release-controlling barrier are formed as separate functional layers, so the upper barrier can be modified independently.

In our previous work, this barrier was tuned by laser microperforation: defined openings were introduced into the face layer after film fabrication [11]. This gives precise local control over individual release pathways, but requires post-processing and is less convenient when permeability must be adjusted over a larger area. Here, we examine a formulation-based alternative. Water-soluble porogens are incorporated into the upper PLGA layer during casting; after hydration, the porogen-rich phase leaches out and increases barrier permeability through pore-like defects and hydrophilic transport pathways. Thus, laser microperforation and porogen incorporation address the same barrier layer in different ways: one creates local apertures, while the other changes the transport properties of the layer more broadly.

Although this strategy is conceptually simple, porogen-mediated barrier control is highly formulation-dependent. Previous controlled-porosity systems show that leachable additives can regulate water ingress and drug transport, but their effect depends strongly on additive distribution within the polymer layer [7,12,13]. However, a water-soluble polymer is not automatically a useful porogen. Its compatibility with the PLGA solution, phase separation during drying, and spatial distribution within the barrier determine whether the final film contains functional transport pathways or only surface defects [14]. Previous studies have also shown that both the identity and loading of water-soluble additives can strongly affect release behavior, film morphology, coating properties, and mechanical performance [15,16]. Similar principles have been applied in biodegradable reservoir systems, where PEG was used to tune the permeability of PLA-based membranes [17,18].

Among hydrophilic excipients, polyethylene glycol (PEG) and polyvinylpyrrolidone (PVP) are the most common candidates for porogen use in polymer films. PEG is widely employed as a hydrophilic modifier. PVP is a long-established pharmaceutical excipient and is frequently used to enhance drug release from polymer matrices [15,16]. Poly(2-ethyl-2-oxazoline) (PEOx), a member of the poly(2-oxazoline) family, has attracted increasing interest as a hydrophilic polymer for biomaterials applications and as a potential alternative to PEG in biomedical formulations [19,20,21,22]. Unlike PEG, PEOx has a polyamide backbone and has been discussed as a polymer with favorable biocompatibility and reduced exposure to some PEG-specific limitations, including oxidative degradation and anti-PEG immune responses. This makes PEOx a promising PEG-alternative polymer with largely unexplored potential as a leachable porogen in antibacterial PLGA films.

Accordingly, this study compares PEG, PVP, and PEOx within the same PLGA barrier of PLACE films intended for local antibacterial delivery. The aim was to determine which additives can form a stable, controlled-permeability barrier without uncontrolled phase separation or burst-dominated release. Morphology, physical stability, model-dye release, vancomycin elution, and MRSA activity were evaluated in sequence. The study positions porogen selection as a critical formulation step for porosity-assisted PLACE coatings and defines a working formulation window for PEOx-containing antibacterial films.

## 2. Materials and Methods

### 2.1. Materials

Poly(glycolic-*co*-lactic acid) (PLGA; PURASORB^®^ PDLG 5010; Corbion N.V., Amsterdam, The Netherlands) with a 50:50 lactide:glycolide molar ratio was used as the main film-forming polymer. Poly(vinyl alcohol) (PVA; molecular weight 72,000 g/mol; degree of hydrolysis 85–89%; AppliChem GmbH, Darmstadt, Germany) was used for the drug-containing layer. Vancomycin sodium salt (Vankorus, LEKKO, Volginsky, Russia) and methylene blue (Sigma-Aldrich, Darmstadt, Germany) were used as model cargoes.

PEG 400 (Ecos-1, Russia), PVP K17 (Macklin, Shanghai, China), and poly(2-ethyl-2-oxazoline) (PEOx; average *M_w_* ∼50,000 Da; PDI 3–4; Macklin, Shanghai, China) were used as the main water-soluble porogens in release experiments. Additional PEG and PVP grades used for preliminary screening included PEG 2000 and PEG 8000 (Macklin, Shanghai, China), PVP with *M_w_* ∼55,000 Da (Sigma-Aldrich, Darmstadt, Germany), and PVP K90 (Ashland, Calvert City, KY, USA). The full list of porogens is provided in Appendix A Table A1.

Acetone and ethyl acetate were purchased from EKOS-1 company (Staraya Kupavna, Russia) and used as received. Other reagents, unless specified separately, were obtained from Macklin and used without further purification. Deionized water was obtained from a Milli-Q water purification system. Phosphate-buffered saline (PBS, 0.1 M; Minimed, Russia) was used as the release medium.

### 2.2. Films Preparation

PLACE films were prepared using the general fabrication workflow described previously for multilayer drug-eluting films, with modifications related to porogen incorporation [10]. In brief, a PLGA-coated polypropylene substrate was used as the support, and the drug-containing PVA matrix was deposited onto the pre-coated surface in the form of snake-patterned domains. A third top layer, consisting of PLGA with the added porogen, was then applied over the structure to form the release-controlling barrier. The resulting multilayer films were dried stepwise and used for further characterization and release experiments without removal from the substrate.

The drug-containing PVA matrix was prepared from the viscous aqueous PVA solution used in the previously established PLACE film workflow [10]. Briefly, PVA was dissolved in DI water under stirring at 90 °C, and the base solution was stored in sealed vials before use. The viscosity of the PVA solution was kept at approximately 350–400 mPa·s, which was suitable for wetting the PLGA-coated substrate and printing continuous snake-patterned domains without breaks. Detailed printing parameters and the software-controlled deposition procedure were described in our previous work [10]. Methylene blue or vancomycin was added directly to the PVA solution before printing. The drug concentrations were 20 mg/mL for methylene blue and 150 mg/mL for vancomycin. These values were selected as technologically processable loading levels that provided measurable release and antibacterial activity without disrupting continuous PVA track printing. After drying, the printed PVA tracks had a height of approximately 16 μm in methylene blue-loaded films and approximately 20 μm in vancomycin-loaded films.

The upper PLGA barrier was prepared from a 7.5 wt.% PLGA solution in an ethyl acetate/acetone mixture. Ethyl acetate was used as the main solvent as a less hazardous alternative to chlorinated solvents. Acetone was added at 10 vol.% to improve solution stability during storage, since PLGA solutions in pure ethyl acetate tended to undergo gradual gelation. The selected solvent composition and polymer concentration gave a solution viscosity of approximately 60–80 mPa·s, as measured at 25 °C with a ROTAVISC lo-vi Complete viscometer (IKA, Staufen, Germany). This viscosity range was suitable for blade coating: the solution remained sufficiently flowable for uniform spreading while retaining enough viscosity to form a continuous wet film.

In the present study, water-soluble porogens were introduced into the upper PLGA barrier layer as additional components of this polymer solution. The porogen content was calculated as +n wt.% relative to the dry PLGA mass in the barrier layer. This convention was used to express the additive fraction in the final dry PLGA-based film rather than in the casting solution. Porogens were added at 5, 10, or 20 wt.%, depending on the experimental series. PEG, PVP, and PEOx were evaluated as candidate porogens. All porogens used for the main release experiments were soluble under the selected preparation conditions. Within the studied concentration range, changes in porogen content mainly affected phase distribution, pore-generating morphology, and permeability after hydration, rather than solution processability or the nominal dry thickness defined by the coating gap and PLGA solution concentration.

Films were formed by blade coating. Polypropylene substrates were fixed on a vacuum perforated plate, and the polymer solution was spread over the substrate using an applicator blade moving at a fixed gap. The same coating parameters were used for the base PLGA layer and for the upper PLGA/porogen barrier layer. In both cases, an applicator gap of 150 μm was used, yielding continuous dry PLGA-based films with a thickness of approximately 6–8 μm under the selected solution composition and drying conditions. This thickness range was chosen to obtain films that were durable enough for handling and release testing while still allowing measurable transport through the barrier. The upper barrier layer was cast on a hot plate at 40 °C when required to improve film uniformity and porogen distribution.

### 2.3. Porogen Screening and Morphology Assessment

Different porogen types and concentrations were screened with the aim of obtaining a homogeneous upper barrier film and a reproducible porous microstructure after hydration. The resulting films were first evaluated visually during casting and after drying. Selected samples were then incubated in DI water at 37 °C to dissolve the porogen phase and reveal the resulting porous structure.

Film morphology was examined by scanning electron microscopy (SEM) using Module Sci PicoEye-150 (Module Sci, Daejeon, Republic of Korea) and Quattro ESEM (Thermo Fisher Scientific, Waltham, MA, USA) microscopes. Samples were mounted on conductive carbon tape and sputter-coated with a 5 nm gold layer prior to imaging. SEM images were acquired at accelerating voltages of 3–7 kV. Both film sides were examined. Pore-size distributions were estimated from top-view SEM images using ImageJ 1.54r software. For each analyzed formulation, three regions from two independently prepared film batches were evaluated, and equivalent circular pore diameters were calculated from measured pore areas. Defect-dominated morphologies were not included in pore-size statistics.

### 2.4. Release Studies and Kinetic Analysis

**Figure A1:**
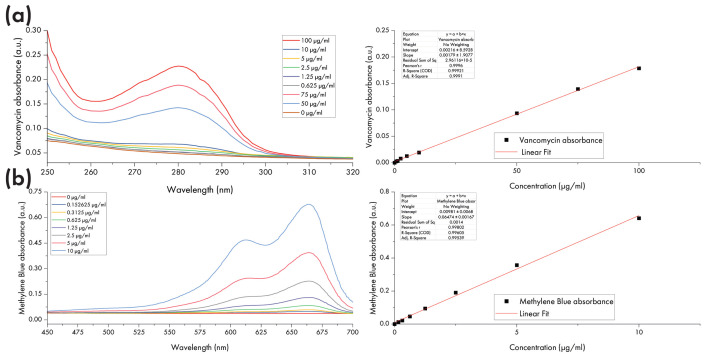
Spectrophotometric calibration used for the quantification of released cargo. (**a**) Absorbance spectra of vancomycin standards in PBS and the corresponding calibration curve at 282 nm. (**b**) Absorbance spectra of methylene blue standards in PBS and the corresponding calibration curve at 664 nm. Linear fitting equations and coefficients of determination are shown on the plots.

Drug release from PLACE films was studied in 5 mL of 0.1 M PBS at 37 °C under constant orbital shaking at 300 rpm. The film area was 2 cm^2^. These conditions were used to maintain bulk mixing of the release medium during sampling intervals. Since the total cargo loading was in the microgram range per sample and several milliliters of release medium were used, saturation of the external medium was not expected to limit release, consistent with the general sink-condition rationale used in in vitro release testing [23,24]. At predetermined time points, the samples were transferred to fresh buffer, and the collected medium was analyzed spectrophotometrically using a Tecan Infinite 200Pro microplate reader and UV-transparent 96-well plates. Calibration curves are provided in the Appendix A Figure A1. Release experiments were performed with 6–8 replicates.

For estimation of the initial cargo loading, six films were prepared using the same PVA printing procedure but without the upper PLGA barrier layer. The cargo was then completely washed out from each sample, and the recovered solution was analyzed spectrophotometrically. The loading was calculated per unit film area and expressed as μg/cm^2^.

Methylene blue-loaded films were used for preliminary screening of porogen-assisted release. Formulations with different porogen compositions in the upper PLGA barrier layer were incubated in PBS, and the released dye was quantified at 664 nm. Cumulative release was calculated relative to the total loading estimated from the sample area.

Vancomycin-loaded films of the same multilayer design were then tested under identical conditions. Vancomycin concentration was determined at 282 nm using the calibration curve provided in the Appendix A Figure A1, and the resulting release profiles were used to compare the selected formulations.

Release profiles were additionally analyzed using DDSolver ver. 1.0 software. Cumulative release data were fitted to commonly used empirical and semi-empirical models, including zero-order, first-order, Higuchi, Korsmeyer–Peppas, Peppas–Sahlin, Weibull, Gompertz, and Hopfenberg models. The fitting was used as a supporting tool for comparing release profiles rather than as the sole basis for mechanistic interpretation. Full fitting results are provided in the Appendix A Table A2, Table A3, Table A4 and Table A5.

### 2.5. Antibacterial Testing

The antibacterial activity of vancomycin-loaded films containing 5 wt.% PEOx was evaluated against methicillin-resistant *Staphylococcus aureus* (MRSA). A clinical MRSA isolate from an internal bacterial collection was used. Before testing, the films were disinfected by UV irradiation for 30 min on each side. Two methods were used for cross-validation: a disk diffusion assay and a modified serial dilution assay.

For the disk diffusion assay, Mueller–Hinton agar was inoculated with a bacterial suspension containing approximately $1.5\times 10^{8}$ CFU/mL of *S. aureus*. The films were placed on the agar surface and incubated for 24 h at 37 °C. The inhibition zone was measured in 10 replicates and expressed as the mean ± SD.

To assess the duration of antibacterial activity during progressive release, the films were first incubated in sterile liquid medium without bacteria for 1–7 days. On each day, the medium was replaced with 5 mL of a fresh *S. aureus* suspension prepared at $10^{3}$, $10^{4}$, $10^{5}$, or $10^{6}$ CFU/mL. After 24 h of contact with the films, broth turbidity was assessed visually, and 0.5 mL aliquots were plated on solid medium using a L-shaped Drigalski spreader for colony evaluation after a further 24 h of incubation at 37 °C. Films preincubated for 6 and 7 days were additionally fragmented before inoculation to assess residual antibiotic retained within the polymer structure.

## 3. Results and Discussion

### 3.1. Porogen-Assisted Permeability Control in PLACE Films

PLACE films were treated as planar reservoir-like systems in which the drug-containing PVA domains are covered by an upper PLGA barrier layer. In this architecture, release is governed by water penetration through the barrier and subsequent cargo dissolution and diffusion. In contrast to our previously reported laser-made openings, which acted as discrete release pathways, the present work focused on porogen-mediated permeability control of the PLGA barrier.

Water-soluble porogens were chosen because they can be added directly to the PLGA solution before casting and then leached out during hydration, forming pores or hydrophilic transport pathways. PEG and PVP were selected as conventional hydrophilic pharmaceutical excipients, while PEOx was included as a biomaterial-oriented alternative to PEG. Solid porogens and particulate templates, including salts, sugars, and gelatin-based particles, have been widely used to generate porous PLGA structures, but they require control over particle dispersion, removal, and pore interconnectivity [25,26,27,28].

**Figure 1:**
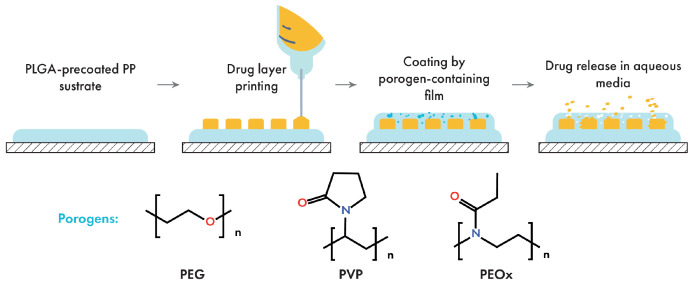
Schematic representation of porogen-assisted PLACE films. Patterned drug-containing domains are printed onto a PLGA-precoated polypropylene substrate and covered with an upper porogen-containing PLGA barrier. Upon immersion in aqueous medium, porogen leaching increases barrier permeability and enables cargo release from the reservoir layer. The chemical repeat units of PEG, PVP, and PEOx are shown as representative porogens.

The main formulation challenge was the incompatibility between hydrophilic porogens and the hydrophobic PLGA matrix. Phase separation during casting can produce surface droplets or large defects instead of a barrier morphology suitable for controlled water ingress and cargo transport. Therefore, candidate porogens were first screened for film-forming behavior and post-hydration morphology. The target structure was a macroscopically uniform barrier layer that, after hydration, developed pore-like features and transport pathways sufficient to increase permeability without uncontrolled film disruption. The general concept of the porogen-assisted PLACE architecture is shown in Figure 1.

### 3.2. Screening of Candidate Porogens for PLGA Barrier Layers

**Figure 2:**
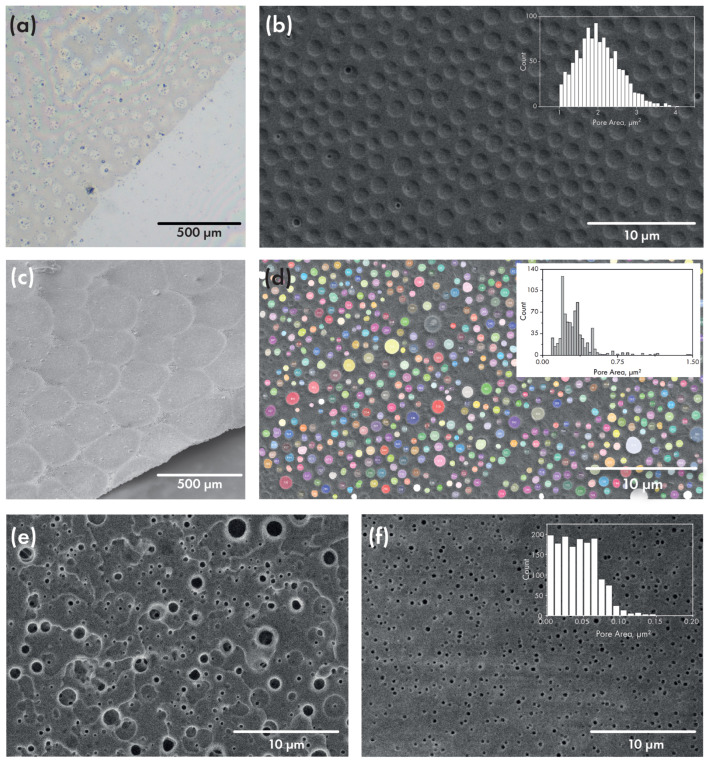
Screening of PEG, PVP, and PEOx as porogens for the upper PLGA barrier layer. (**a**) PEG 2000-containing film after partial immersion in water. (**b**) SEM image of PEG 400-containing film after hydration, with feature-size distribution shown in the inset. (**c**) Phase separation of 10 wt.% PVP K17 on a non-heated plate. (**d**) 10 wt.% PVP K17 film prepared on a heated plate with highlighted PVP-rich clusters and feature-size distribution shown in the inset. (**e**) Canyon-like erosion on the bottom side of a 20 wt.% PEOx film after hydration; pore-size statistics were not calculated for this sample because it was clearly defective. (**f**) Porous morphology of a 5 wt.% PEOx film prepared on a heated plate, with pore-size distribution shown in the inset.

**Figure A2:**
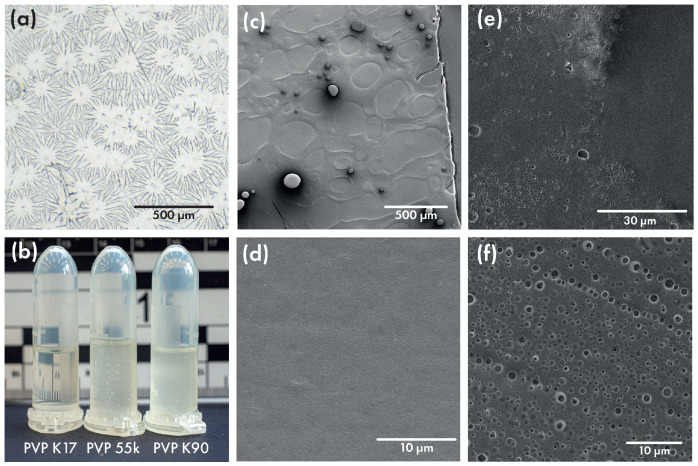
Additional images from preliminary porogen screening. (**a**) Optical image of crystallized PEG 8000. (**b**) Emulsification of PLGA solutions after the addition of 10 wt.% PVP 55k and PVP K90, with a clear mixture obtained for PVP K17 (from left to right: PVP K17, PVP 55k, and PVP K90). (**c**,**d**) Films containing 20 wt.% PVP was prepared on a non-heated plate (phase separation) and on a hot plate, respectively. (**e**) Non-uniform distribution of PEOx in a film prepared on a non-heated plate. (**f**) Comb-like alignment of droplet tracks in a film containing 10 wt.% PEOx.

The first step was to identify water-soluble additives capable of forming a stable porogen-containing PLGA barrier layer. The desired outcome was not only visual film uniformity after casting, but also the formation of a fine porous structure after hydration. Therefore, the candidate porogens were evaluated based on solution stability, film-forming behavior, phase separation during drying, and post-hydration morphology. Representative examples of the screening results are shown in Figure 2, and additional screening images are provided in Appendix A Figure A2.

PEG was first evaluated as a conventional hydrophilic porogen. However, PEG grades with higher molecular weight showed poor compatibility with PLGA during film casting. PEG 2000 and PEG 8000 formed large irregular clusters, in some cases reaching hundreds of micrometers, which floated on the PLGA surface. Their size and distribution were poorly reproducible and did not correlate clearly with the amount of additive, indicating strong phase separation (Figure 2a). Heating the casting plate to 40–45 °C slightly reduced the cluster size, but did not provide a uniform film. PEG 400 showed better processability. At ambient temperature, phase separation was still observed, whereas casting on a heated plate allowed visually homogeneous films to be obtained at 10 wt.% PEG 400. However, higher PEG 400 content again produced large surface droplets. After immersion in water, SEM images revealed semicircular recesses left by PEG-rich domains (Figure 2b). This indicates that PEG 400 was localized mainly near the film surface; after hydration, it left shallow surface defects rather than pathways capable of supporting gradual release through the barrier. Therefore, PEG was excluded from further release studies. This behavior is consistent with previous PLGA thin-film studies, where PEG additives were shown to affect drug release depending on PEG molecular weight, concentration, phase separation, and distribution within the PLGA matrix [14,18].

The compatibility of PVP depended more strongly on molecular weight. For PVP with *M_w_* ∼55,000 Da and the high-molecular-weight K90 grade, poor compatibility with PLGA was already evident at the solution stage: addition of 10 wt.% of these grades caused visible stratification of the PLGA solution. PVP K90 also markedly increased viscosity, further complicating film casting. In contrast, the lower-molecular-weight PVP K17 formed clear and stable mixtures without a noticeable change in viscosity and was therefore selected for film preparation.

PVP K17 films were prepared both at ambient temperature and on a heated plate. Without heating, phase separation remained pronounced: at 10 wt.% PVP K17, hexagonal PVP-rich regions were observed, while 20 wt.% led to almost complete phase separation. Heating improved the film uniformity, and the most homogeneous samples were obtained when casting was performed on a hot plate. Under SEM imaging, small PVP-rich domains of approximately 0.2–2 μm became visible, presumably because local heating of the polymer film by the electron beam enhanced contrast between the PVP and PLGA phases (Figure 2c,d). Despite improved processing, PVP K17 did not produce a completely uniform morphology. After hydration, the films developed large, flat, crater-like features, consistent with local enrichment of the PVP-rich phase near the film surface. Such morphology is unlikely to represent a fully interconnected pore network across the barrier layer, but it can still increase water uptake, swelling, and local permeability of the PLGA film. Therefore, PVP K17 was retained as the main conventional pore-forming candidate for further release experiments.

PEOx showed different behavior. Because only one PEOx grade was available, poly(2-ethyl-2-oxazoline) with an average molecular weight of about 50,000 was tested at several concentrations. At loadings of 5 and 10 wt.%, PEOx-containing films cast at 40 °C developed well-defined porosity after hydration. In top-view SEM images, the pores appeared mostly rounded and were distributed across the observed film surface, typically measuring about 100–300 nm. Relative to PVP, the morphology produced by PEOx appeared less dominated by large surface craters, which points to a more favorable distribution of the porogen-rich phase throughout the barrier layer. Raising the PEOx content to 10 wt.% broadened the pore population, yielding both small pores and larger features of roughly 1–3 μm. Partial alignment of porogen-derived domains along the coating direction was also observed on the upper surface. This comb-like pattern was most likely the result of the deformation and breakup of the PEOx-rich phase during blade coating. At 20 wt.% PEOx, the system lost structural stability: following hydration, the lower face of the film exhibited canyon-like erosion features, signaling coalescence of larger porogen-rich domains and a loss of controlled phase distribution (Figure 2e,f).

Taken together, the primary screening indicated that not all hydrophilic additives were appropriate for porosity-mediated tuning of the PLGA barrier. PEG was therefore excluded from further release studies, as its phase separation primarily produced surface-localized defects and did not provide the type of barrier opening required for gradual, controlled release. The high-molecular-weight PVP grades were also ruled out on account of their poor compatibility with the PLGA solution. PVP K17 and PEOx were therefore retained as the main working porogens. PVP K17 represented the more conventional pore-forming excipient, whereas PEOx produced a distinct porous morphology and was further evaluated as a biomaterial-oriented alternative to PEG.

### 3.3. Methylene Blue Release from PVP- and PEOx-Containing Films

Methylene blue served as a model cargo for the first comparison of porogen-assisted release from PLACE films. This dye is well suited to the task: its release can be tracked spectrophotometrically at 664 nm and is also readily observed by eye during the experiment. Together with its moderate aqueous solubility and stability, these features allow differences in barrier permeability to be resolved even when release proceeds relatively quickly. Calibration curves for methylene blue are given in the Appendix A Figure A1. Initial release experiments were carried out on films incorporating 10 or 20 wt.% PVP K17 in the upper PLGA barrier layer, with a total methylene blue loading of 122 ± 13 μg/cm^2^. Prior to release testing, the films exhibited smooth and visually homogeneous surfaces. After prolonged SEM exposure, small PVP-rich domains ranging from sub-micrometer to a few micrometers became discernible, presumably due to localized heating of the polymer film under the electron beam.

**Figure 3:**
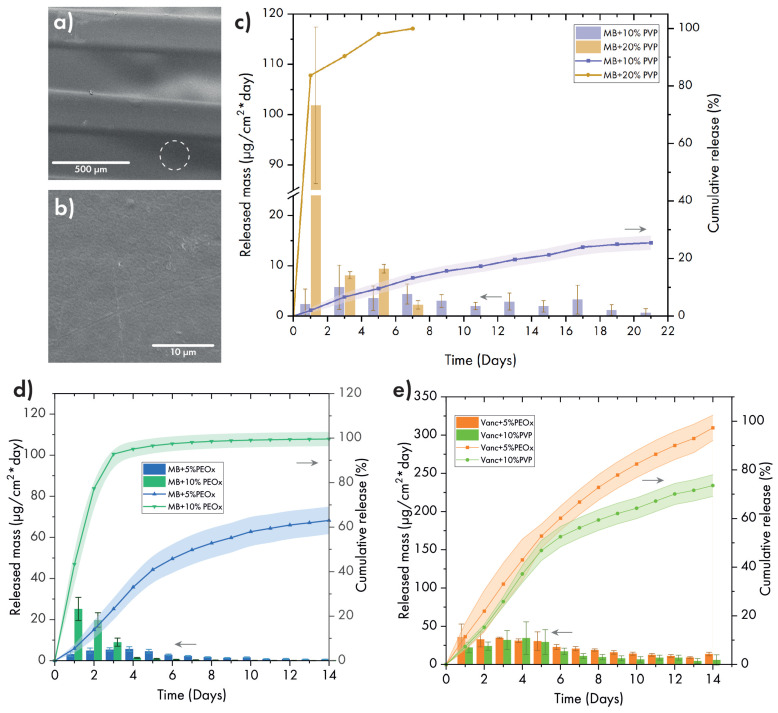
Methylene blue and vancomycin release from porogen-containing PLACE films. (**a**,**b**) SEM images of films containing 10 wt.% PVP K17 before release, showing an initially smooth surface with small PVP-rich domains visible under prolonged SEM exposure. (**c**) Daily and cumulative release profiles of methylene blue from films containing 10 and 20 wt.% PVP K17 in the upper PLGA barrier layer. (**d**) Daily and cumulative release profiles of methylene blue from films containing 5 and 10 wt.% PEOx in the upper PLGA barrier layer. (**e**) Daily and cumulative release profiles of vancomycin from selected formulations containing 10 wt.% PVP K17 and 5 wt.% PEOx.

The SEM images of the PVP-containing films before release and the release profiles for methylene blue and vancomycin are summarized in Figure 3. Incorporation of 10 wt.% PVP K17 yielded an extended-release profile, with roughly 30% of the loaded methylene blue released over the course of three weeks. Day-to-day release values varied markedly between samples, which we attribute to the uneven spatial distribution of PVP-rich domains within the barrier layer. Given the limited surface area of each sample, local variations in the number and dimensions of these domains locally affect the measured release rate.

**Figure 4:**
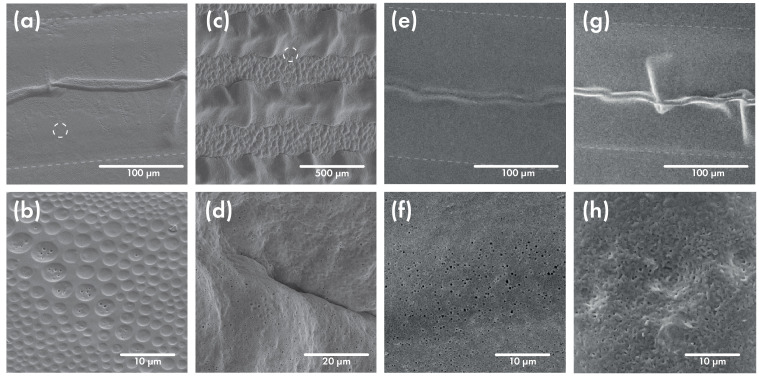
SEM images of porogen-containing PLACE films after 14 days of release in PBS. (**a**,**b**) Films containing 10 wt.% PVP K17. (**c**,**d**) Films containing 20 wt.% PVP K17. (**e**,**f**) Films containing 5 wt.% PEOx. (**g**,**h**) Films containing 10 wt.% PEOx. Dashed lines indicate the approximate boundaries of the drug-loaded stripes.

Raising the PVP content to 20 wt.% altered release behavior markedly. Under these conditions, around 80% of the dye was released during the first 24 h, pointing to a burst-dominated profile (Figure 3c). This finding is consistent with the post-release morphology shown in Figure 4: films with 20 wt.% PVP displayed numerous micron-sized craters in the range of approximately 1–8 μm, corresponding to leached-out PVP-rich domains, with the larger craters additionally featuring submicron pores. Such a morphology reflects more pronounced phase separation and the development of large transport defects in the PLGA barrier, accounting for the rapid release of the dye. This agrees with the general view that leachable additives and polymer blending can regulate water ingress and drug transport, but the final release behavior is determined by additive miscibility, phase distribution, and the morphology of the hydrated polymer barrier rather than by hydrophilicity alone [12,16]. Films loaded with 10 wt.% PVP, by contrast, preserved a more intact reservoir architecture after three weeks of incubation. They appeared swollen and rough, in line with water ingress into the polymer matrix promoted by hydrophilic PVP domains. Large crater-like defects, however, were not the predominant feature of these samples, and more than 70% of the initial dye load remained in the reservoirs at the end of the experiment. The 10 wt.% PVP K17 formulation therefore offered a more gradual release regime, whereas 20 wt.% PVP caused excessive opening of the barrier and was unsuitable for controlled prolonged release.

Release kinetics served as a supporting tool for describing the 10 wt.% PVP K17 profile. The Korsmeyer–Peppas model gave a stable fit with a release exponent close to 0.6, consistent with anomalous, non-Fickian transport. This indicates that methylene blue release from these films was not governed by simple diffusion alone, but involved diffusion through hydrated pathways together with relaxation or swelling of the polymer structure.

The Peppas–Sahlin model gave a formally higher goodness of fit, but the fitted Fickian coefficient $k_{1}$ was negative. Since both terms in this model are expected to be non-negative, this result was considered physically unstable and was not used for mechanistic interpretation. In the present system, the early release stage includes water ingress into the PLGA barrier, porogen leaching, and hydration of the underlying PVA reservoir. These processes can produce a lag phase before continuous transport pathways are established, and the Peppas–Sahlin model appears to approximate this profile through compensation of its two terms rather than through a physically meaningful separation of Fickian and relaxational contributions. The Peppas–Sahlin fit is therefore retained in Appendix A only for completeness, together with the Hopfenberg fit, whose parameters were also unstable. Full fitting results are given in Table A2, Table A3, Table A4 and Table A5.

Since increasing PVP content accelerated release mainly at the cost of burst behavior and stronger morphological disruption, PEOx was evaluated as an alternative porogen with potentially different distribution within the PLGA barrier. Prior to incubation, PEOx-containing films appeared visually smooth, with no macroscopic defects, droplets, or signs of phase separation. The comparison between 5 and 10 wt.% PEOx was therefore based on release behavior and post-release morphology. PEOx altered the release behavior to a greater extent than PVP K17 (Figure 3d). At 5 wt.%, methylene blue release was already faster than from films loaded with 10 wt.% PVP. The 5 wt.% PEOx formulation, however, did not produce a sharp burst. Its release curve was noticeably smoother than that of the 20 wt.% PVP system, in which the majority of the dye was released within the first 24 h. Increasing the PEOx content to 10 wt.% shifted the system to a much faster regime. Most of the dye was released within the first several days, after which the curve gradually approached a plateau. This profile was not identical to the one-day burst observed for 20 wt.% PVP; rather, it suggested a short opening stage followed by rapid depletion of the accessible dye fraction.

Kinetic fitting was performed for the 10 wt.% PEOx formulation as the most pronounced example of fast PEOx-mediated release. The Korsmeyer–Peppas model was poorly suited for this profile: it gave low goodness-of-fit values and a very low exponent (n=0.178±0.034), so this fit was not used for mechanistic interpretation. The first-order model fit the curve better ($k_{1}=0.704\pm 0.121$), which agrees with rapid release after hydration and opening of the barrier layer. The Hopfenberg model also fitted some samples reasonably well, but the fitted parameters were unstable and physically unrealistic. Thus, the 10 wt.% PEOx profile is better described as rapid release from a progressively hydrated and highly permeable barrier, rather than as simple diffusion-controlled transport.

SEM images collected after the release experiment showed clear differences between the two PEOx concentrations (Figure 4). Films with 5 wt.% PEOx contained visible pores, and the drug-loaded stripes appeared deflated in the regions where methylene blue had been released. However, the polymer layer did not show pronounced swelling or foaming. In contrast, 10 wt.% PEOx produced a much more porous surface after incubation. Despite this developed porosity, the morphology remained more coherent than in films with 20 wt.% PVP, where large crater-like defects and strong swelling were observed. This indicates that PEOx increased barrier permeability efficiently, but did not produce the same type of large-scale surface collapse as excessive PVP loading.

Overall, PEOx acted as a stronger permeability modifier than PVP K17. The 10 wt.% PEOx formulation provided fast and nearly complete dye release, but was considered too fast for the intended antibacterial formulation. The 5 wt.% PEOx formulation was more balanced: it increased early release compared with 10 wt.% PVP while avoiding the abrupt burst and morphological disruption associated with 20 wt.% PVP. For this reason, 5 wt.% PEOx was selected for subsequent vancomycin loading. Although PEOx was used here as a leachable porogen rather than as a permanent stealth or stabilizing component, its selection is also consistent with the broader interest in poly(2-oxazoline)s as PEG-alternative hydrophilic polymers in biomedical formulations [22].

### 3.4. Vancomycin Release from Selected Porogen-Containing Films

Vancomycin was used as the antibacterial cargo for the MRSA experiments. It is active against Gram-positive bacteria, methicillin-resistant *Staphylococcus aureus* included, and was therefore considered suitable for testing local antibacterial release from porogen-modified PLACE films.

Two formulations from the methylene blue series were taken forward for vancomycin loading: films with 10 wt.% PVP K17 in the upper PLGA barrier, and films with 5 wt.% PEOx. Cumulative and daily release profiles are given in Figure 3e. The initial vancomycin loading was 312.0 ± 27.0 μg/cm^2^. Since the tested sample area was 2 cm^2^, this corresponded to approximately 624 ± 54 μg of vancomycin per sample.

Vancomycin release continued over two weeks for both formulations, but the two profiles were not the same. The 10 wt.% PVP K17 films released the drug more slowly. Daily values reached their maximum during the first 3–4 days and then dropped. By day 14, the cumulative curve had reached roughly three-quarters of the loaded amount.

The 5 wt.% PEOx films released vancomycin faster. The first days of incubation showed appreciable sample-to-sample variability, after which the cumulative curve became more regular and approached near-complete release by day 14. We attribute the early variability to the porogen-leaching stage. Before stable transport pathways are formed, small differences in the local PEOx distribution can affect water penetration through the PLGA barrier and the onset of vancomycin diffusion. Once the barrier is hydrated and the main pathways are open, release becomes more regular. The same trend was already seen with methylene blue, where PEOx had acted as a stronger permeability modifier than PVP K17. In absolute units, the 10 wt.% PVP K17 films released approximately 22–34 μg/cm^2^/day during the first several days, followed by a decrease to approximately 5–9 μg/cm^2^/day by the end of the experiment. For the 5 wt.% PEOx films, the early daily release was higher, approximately 30–36 μg/cm^2^/day, and then decreased to approximately 12–16 μg/cm^2^/day by days 11–14. By day 14, the cumulative released amount was approximately 220–235 μg/cm^2^ for the 10 wt.% PVP K17 films and approximately 300–312 μg/cm^2^ for the 5 wt.% PEOx films.

Kinetic fitting was used as a supporting descriptive tool. For 10 wt.% PVP K17, the Korsmeyer–Peppas model gave a stable fit with n=0.625, indicating anomalous, non-Fickian transport. Thus, vancomycin release from this formulation was not described as simple diffusion, but as a process involving diffusion through hydrated pathways together with hydration and relaxation of the polymer structure. The Peppas–Sahlin and Weibull models gave higher formal goodness-of-fit values for the 10 wt.% PVP K17 profile. However, the Peppas–Sahlin fit produced physically unstable coefficients with large opposite signs, and was therefore not used for mechanistic interpretation. The Hopfenberg model was also tested, but its fitted parameters were unrealistic and were not interpreted mechanistically. We therefore describe the PVP K17 profile as prolonged non-Fickian release without assigning it to a single mechanistic model.

For 5 wt.% PEOx, the Korsmeyer–Peppas model gave a reasonable semi-empirical description, with n=0.682±0.121, also indicating anomalous transport. The Weibull model gave the best empirical fit in this series ($R^{2}=0.988$–0.998, β=1.264±0.217), consistent with progressive non-burst release. The Hopfenberg model was also tested for the PEOx profiles, but its parameters were unstable and were not used for interpretation.

The vancomycin data, therefore, showed two different functional regimes. The 10 wt.% PVP K17 films gave a slower, more sustained release suitable for prolonged drug retention. The 5 wt.% PEOx films, despite the lower porogen loading, gave higher early output and near-complete release within two weeks, with no abrupt burst and no visible loss of film integrity at the end of the experiment. On this basis, 5 wt.% PEOx was used in the subsequent antibacterial experiments against MRSA.

### 3.5. Antibacterial Activity of 5 wt.% PEOx Films Against MRSA

Based on the release profiles described above, the 5 wt.% PEOx formulation was selected for antibacterial testing against MRSA. PEOx alone at 100 μg/mL was used as a control and showed no detectable antibacterial activity. We therefore attribute the biological effect of the films to vancomycin release.

Before discussing the antibacterial results, we note one feature of the assay. Each ∼2 cm^2^ sample was incubated in 5 mL of bacterial suspension, which corresponds to a comparatively large medium volume per film area. The local concentration of vancomycin around the film was therefore lower than what would be present in a tissue-contacting setting, where only a thin fluid layer surrounds the coating. The activity reported here should therefore be regarded as a conservative estimate of local antibacterial performance.

**Figure 5:**
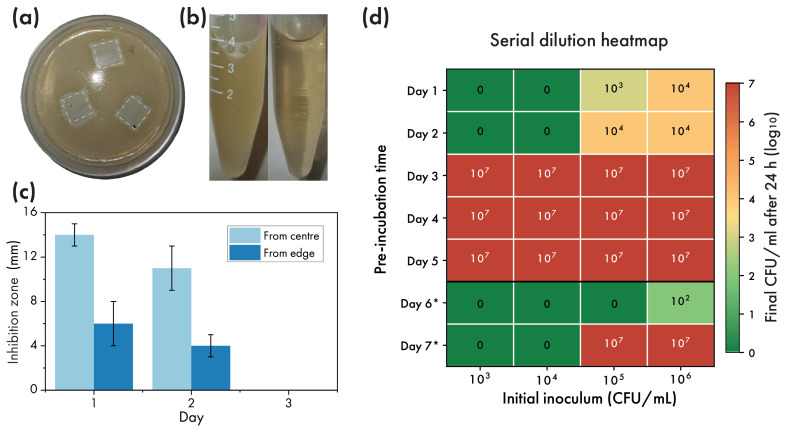
Antibacterial performance of vancomycin-loaded PLACE films containing 5 wt.% PEOx against MRSA. (**a**) Representative disk diffusion test on Mueller–Hinton agar inoculated with *S. aureus*. (**b**) Comparison of bacterial suspensions after incubation: turbid samples indicate active bacterial growth, whereas transparent samples indicate growth suppression. (**c**) Quantification of inhibition zones measured from the center of the sample and from the substrate edge. (**d**) Heatmap of final bacterial titers obtained in the modified serial dilution assay after incubation with intact or fragmented films. Asterisks mark days on which the films were mechanically fragmented before inoculation to evaluate residual antibiotic retained inside the polymer matrix.

The disk diffusion assay confirmed early release of active vancomycin from the films. After 24 h of incubation, the inhibition zone measured 14 ± 1 mm from the center of the sample (6 ± 2 mm from the substrate edge). On the second day, the zone was still clearly visible but had narrowed to 11 ± 2 mm (4 ± 1 mm from the edge), corresponding to a ∼30% reduction (Figure 5c). By the third day, the bacterial lawn formed a dense contour directly around the substrate, and reliable measurement was no longer feasible. This is consistent with the release data: the main freely diffusible fraction of vancomycin was delivered during the first days, after which the local antibiotic flux dropped.

The disk diffusion assay confirmed early release of active vancomycin from the films (Figure 5a,c). During the first two days, the films exerted a pronounced effect against MRSA. At an initial inocula of $10^{3}$–$10^{4}$ CFU/mL, the effect was primarily bactericidal: the post-incubation titer dropped to zero colonies, indicating complete elimination. At higher inocula of $10^{5}$–$10^{6}$ CFU/mL, the films did not fully sterilize the samples but markedly suppressed bacterial growth. Final titers typically fell within the $10^{3}$–$10^{4}$ CFU/mL range, pointing to a combined bactericidal and bacteriostatic action. From the third day onward, plating more frequently produced confluent growth at $10^{6}$–$10^{7}$ CFU/mL across all starting inocula. The amount of vancomycin released from intact films into 5 mL of medium was no longer sufficient to fully suppress MRSA. Even so, the broth did not always reach the turbidity of the untreated bacterial control (Figure 5b). We read this as residual vancomycin release continuing to limit bacterial expansion, although the released amount was no longer sufficient to prevent survival and growth. The films, therefore, gave strong early activity, followed by weaker bacteriostatic suppression at later time points.

We then asked whether vancomycin still remained inside the films after their direct antibacterial effect had declined. Films preincubated for 6 and 7 days were mechanically fragmented before bacterial challenge. Antibacterial activity reappeared after fragmentation. On day 6, fragmented films reduced all four inocula to ≤100 CFU/mL, with complete elimination at $10^{3}$–$10^{4}$ CFU/mL and a strong bacteriostatic effect at $10^{5}$–$10^{6}$ CFU/mL. On day 7, activity at the low inocula was retained, while the higher inocula reached confluent growth. A non-negligible amount of vancomycin, therefore, remained within the polymer structure at late time points but was not readily accessible from the intact film. The decline of antibacterial activity after several days reflected not only drug depletion, but also the limited availability of the residual drug trapped within the film.

The antibacterial experiments thus identify the 5 wt.% PEOx vancomycin-loaded PLACE films as a short-term, high-output antibacterial system. A strong effect against MRSA was observed during the first two days, with bactericidal activity at the lower inocula and a substantial titer reduction at the higher inocula, even under the conservative dilution conditions of the assay. Activity then declined to residual suppression. The fragmentation experiments, however, showed that vancomycin remained within the polymer matrix beyond the time at which the intact films had lost their effect. We consider these observations to be in agreement with the release profile and supportive of the use of 5 wt.% PEOx as a porogen for early local antibacterial exposure.

## 4. Conclusions

This study extends the previously reported PLACE platform from discrete-orifice-based release to porosity-assisted barrier control. Instead of forming defined transport routes via laser microperforation, release was tuned by incorporating and leaching water-soluble porogens in the upper PLGA barrier layer. The porogen in this design is not just a soluble additive: it acts as a structure-forming component that determines water ingress, pore architecture, and cargo output.

The comparison of PEG, PVP, and PEOx showed that water-soluble polymers are not interchangeable in this role. Their behavior in the same PLGA matrix differed considerably during casting, hydration, and release. PEG mainly produced surface-localized defects rather than a controlled-permeability barrier morphology suitable for gradual transport under the studied conditions, and high-molecular-weight PVP grades were limited by poor solution compatibility. PVP K17 was the most workable conventional porogen. At 10 wt.%, it produced sustained release, whereas higher loading shifted the system toward burst-dominated behavior and stronger morphological disruption.

Use of PEOx provided a different formulation window. At low loading, it yielded a more pronounced increase in permeability than PVP K17 while preserving film integrity. In vancomycin-loaded films, 5 wt.% PEOx enabled higher early output and near-complete release over two weeks, and this formulation was therefore taken into the antibacterial evaluation. The MRSA experiments confirmed a short-term, high-output antibacterial profile, with bactericidal activity at lower inocula and substantial titer reductions at higher inocula during the first days.

We therefore consider PEOx a suitable PEG-alternative porogen for early local antibacterial exposure from PLACE films, while PVP K17 remains better suited to slower sustained-release designs. The results indicate that porogen selection can be used as a formulation tool to program the release regime of multilayer antibacterial coatings without changing the drug-loaded reservoir itself.

## Data Availability

The original contributions presented in this study are included in the article. Further inquiries can be directed to the corresponding authors.

## Abbreviations

The following abbreviations are used in this manuscript:

PLACE	Printed Layered Adjustable Cargo Encapsulation
PLGA	Poly(glycolic-*co*-lactic acid)
PLA	Poly(lactic acid)
PVA	Poly(vinyl alcohol)
PEG	Polyethylene glycol
PVP	Polyvinylpyrrolidone
PEOx	Poly(2-ethyl-2-oxazoline)
MB	Methylene blue
MRSA	Methicillin-resistant *Staphylococcus aureus*
PBS	Phosphate-buffered saline
DI water	Deionized water
SEM	Scanning electron microscopy
CFU	Colony-forming units
MW	Molecular weight
PDI	Polydispersity index
SD	Standard deviation
AIC	Akaike Information Criterion
MSC	Model Selection Criterion

## Appendix A

**Table A1:** Water-soluble porogens evaluated during preliminary screening.

Porogen	Grade/Molecular Weight	Supplier	Role in the Study
PEG 400	$M_{w}$ 400 Da	ECOS-1, Staraya Kupavna, Russia	Preliminary screening; selected PEG grade for SEM evaluation
PEG 2000	$M_{w}$ 2000 Da	Macklin, Shanghai, China	Preliminary screening; excluded due to phase separation
PEG 8000	$M_{w}$ 8000 Da	Macklin, Shanghai, China	Preliminary screening; excluded due to phase separation
PVP K17	K17; low-molecular-weight grade, *M_w_* ∼10,000 Da	Macklin, Shanghai, China	Main PVP grade used for release studies
PVP 55k	*M_w_* ∼55,000 Da	Sigma-Aldrich, Darmstadt, Germany	Preliminary screening; excluded due to solution stratification
PVP K90	K90; high-molecular-weight grade, *M_w_* ∼1,000,000–1,500,000 Da	Ashland, Calvert City, KY, USA	Preliminary screening; excluded due to stratification and high viscosity
PEOx	*M_w_* ∼50,000 Da; PDI 3–4	Macklin, Shanghai, China	Main PEOx grade used for release studies

### Fitting Data

Release curves were fitted using DDSolver ver. 1.0 as described in the Section 2. The fitting results are provided in the Appendix for comparison of release profiles. Mechanistic interpretation was based not only on goodness of fit, but also on the physical plausibility and stability of the fitted parameters.

**Table A2:** Kinetic fitting summary for methylene blue release from films containing 10 wt.% PVP K17.

Model	Key Fitted Parameters	$R_{\mathrm{obs-pre}}$	$R^{2}$	MSE	${\mathrm{MSE}}_{\mathrm{root}}$	AIC	MSC
Peppas–Sahlin	$k_{1}=-14\pm 33$; $k_{2}=19\pm 25$; m=0.43±0.33	0.993	0.985	7.22	2.68	61.6	3.74
Korsmeyer–Peppas	$k_{KP}=10\pm 7$; n=0.61±0.12	0.979	0.955	20.6	4.47	73.7	2.81
First-order	–	0.984	0.954	20.2	4.34	71.4	2.98
Hopfenberg	–	0.983	0.954	22.1	4.53	73.4	2.83
Higuchi	–	0.983	0.929	27.2	5.22	77.2	2.53
Hixson–Crowell	–	0.974	0.912	39.8	5.95	79.0	2.40
Zero-order	–	0.942	0.738	122	10.1	91.9	1.40

Data are presented as mean fitting outputs for the analyzed release profile. The Peppas–Sahlin parameters were considered physically unstable because the fitted Fickian coefficient was negative; this model was not used for mechanistic interpretation.

**Table A3:** Kinetic fitting summary for methylene blue release from films containing 10 wt.% PEOx.

Model	Key Fitted Parameters	$R_{\mathrm{obs-pre}}$	$R^{2}$	MSE	${\mathrm{MSE}}_{\mathrm{root}}$	AIC	MSC
Korsmeyer–Peppas	$k_{KP}=66.8\pm 5.6$; n=0.178±0.034	0.818	0.668	87.0	9.20	100.5	0.82
First-order	$k_{1}=0.70\pm 0.12$	0.987	0.947	14.6	3.51	70.3	2.97
Hopfenberg	$k_{HB}=0.14\pm 0.19$; n=477±826	0.990	0.966	9.50	2.88	66.6	3.24

Values are shown as mean ± SD for fitted parameters and as mean goodness-of-fit values across individual samples. Hopfenberg parameters were considered physically unstable and were not used for mechanistic interpretation.

**Table A4:** Kinetic fitting summary for vancomycin release from films containing 10 wt.% PVP K17.

Model	Key Fitted Parameters	$R_{\mathrm{obs-pre}}$	$R^{2}$	MSE	${\mathrm{MSE}}_{\mathrm{root}}$	AIC	MSC
Korsmeyer–Peppas	$k_{KP}=15.1$; n=0.625	0.976	0.950	25.2	5.02	83.9	2.70
Peppas–Sahlin	$k_{1}=-422$; $k_{2}=426$; m=0.051	0.995	0.990	7.21	2.69	72.9	3.86
Weibull	α=8.31; β=0.943	0.992	0.984	10.2	3.19	77.2	3.57
Hopfenberg	$k_{HB}\approx 0$; n=2592	0.992	0.983	11.1	3.33	78.5	3.49

Fitting was performed for the averaged release profile. Peppas–Sahlin and Hopfenberg parameters were not used for mechanistic interpretation because of physically unstable coefficient combinations. In particular, the Peppas–Sahlin fit produced a negative Fickian coefficient, and the Hopfenberg fit produced an unrealistic value of the exponent parameter.

**Table A5:** Kinetic fitting summary for vancomycin release from films containing 5 wt.% PEOx.

Model	Key Fitted Parameters	$R_{\mathrm{obs-pre}}$	$R^{2}$	MSE	${\mathrm{MSE}}_{\mathrm{root}}$	AIC	MSC
Korsmeyer–Peppas	$k_{KP}=17\pm 5$; n=0.68±0.12	0.987–0.996	0.971–0.991	5.24–27.4	2.29–5.23	62.0–85.1	3.25–4.45
Weibull	α=11±6; β=1.26±0.22	0.995–0.999	0.988–0.998	1.97–11.4	1.40–3.38	48.3–72.9	4.16–5.71
Hopfenberg	$k_{HB}=0.05\pm 0.03$; n=13±23	0.994–1.000	0.984–0.999	0.62–14.9	0.79–3.86	32.0–76.6	3.86–6.87

Fitted parameters are presented as mean ± SD. Goodness-of-fit values are shown as ranges across individual samples. Hopfenberg parameters were considered physically unstable and were not used for mechanistic interpretation.

## References

[1] Shukla A., Fleming K.E., Chuang H.F., Chau T.M., Loose C.R., Stephanopoulos G.N., Hammond P.T. (2010). Controlling the Release of Peptide Antimicrobial Agents from Surfaces. Biomaterials.

[2] Toledano-Osorio M., Vallecillo C., Vallecillo-Rivas M., Manzano-Moreno F.J., Osorio R. (2022). Antibiotic-Loaded Polymeric Barrier Membranes for Guided Bone/Tissue Regeneration: A Mini-Review. Polymers.

[3] Mordovina E.A., Plastun V.O., Abdurashitov A.S., Proshin P.I., Raikova S.V., Bratashov D.N., Inozemtseva O.A., Goryacheva I.Y., Sukhorukov G.B., Sindeeva O.A. (2022). “Smart” Polylactic Acid Films with Ceftriaxone Loaded Microchamber Arrays for Personalized Antibiotic Therapy. Pharmaceutics.

[4] Blanco A.F., Lou G., Pensado-López A., Ummarino A., Andón F.T., Crecente-Campo J., Alonso M.J. (2025). Controlled co-delivery of anti-inflammatory drugs from bilayer polymer films coating a meniscus implant. Drug Deliv. Transl. Res..

[5] Bryaskova R., Philipova N., Bakov V., Georgiev N. (2025). Innovative Antibacterial Polymer Coatings. Appl. Sci..

[6] Olamide Ishola B., Rahaman K.A., Razzak S.A., Rumon M.M.H., Shakil M.S., Uddin S. (2026). Advanced mechanisms of polymer-based drug delivery systems for clinical applications. RSC Pharm..

[7] Zentner G.M., Rork G.S., Himmelstein K.J. (1985). The Controlled Porosity Osmotic Pump. J. Control. Release.

[8] Kaunisto E., Marucci M., Borgquist P., Axelsson A. (2011). Mechanistic Modelling of Drug Release from Polymer-Coated and Swelling and Dissolving Polymer Matrix Systems. Int. J. Pharm..

[9] Almoshari Y. (2022). Osmotic Pump Drug Delivery Systems—A Comprehensive Review. Pharmaceuticals.

[10] Proshin P.I., Abdurashitov A.S., Sindeeva O.A., Ivanova A.A., Sukhorukov G.B. (2022). Additive Manufacturing of Drug-Eluting Multilayer Biodegradable Films. Polymers.

[11] Abdurashitov A.S., Proshin P.I., Sindeeva O.A., Sukhorukov G.B. (2022). Laser Microperforation Assisted Drug-Elution from Biodegradable Films. Pharmaceutics.

[12] Ghasemiyeh P., Mohammadi-Samani S. (2021). Polymers Blending as Release Modulating Tool in Drug Delivery. Front. Mater..

[13] Pöttgen S., Mazurek-Budzyńska M., Wischke C. (2025). The role of porosity in polyester microparticles for drug delivery. Int. J. Pharm..

[14] Huang C.L., Steele T.W., Widjaja E., Boey F.Y., Venkatraman S.S., Loo J.S. (2013). The influence of additives in modulating drug delivery and degradation of PLGA thin films. NPG Asia Mater..

[15] Rohera B.D., Parikh N.H. (2002). Influence of Type and Level of Water-Soluble Additives on Drug Release and Surface and Mechanical Properties of Surelease Films. Pharm. Dev. Technol..

[16] Yang M., Xie S., Li Q., Wang Y., Chang X., Shan L., Sun L., Huang X., Gao C. (2014). Effects of Polyvinylpyrrolidone Both as a Binder and Pore-Former on the Release of Sparingly Water-Soluble Topiramate from Ethylcellulose Coated Pellets. Int. J. Pharm..

[17] Jonnalagadda S., Robinson D.H. (2000). A Bioresorbable, Polylactide Reservoir for Diffusional and Osmotically Controlled Drug Delivery. AAPS PharmSciTech.

[18] Steele T.W., Huang C.L., Widjaja E., Boey F.Y., Loo J.S., Venkatraman S.S. (2011). The effect of polyethylene glycol structure on paclitaxel drug release and mechanical properties of PLGA thin films. Acta Biomater..

[19] Lorson T., Lübtow M.M., Wegener E., Haider M.S., Borova S., Nahm D., Jordan R., Sokolski-Papkov M., Kabanov A.V., Luxenhofer R. (2018). Poly(2-oxazoline)s Based Biomaterials: A Comprehensive and Critical Update. Biomaterials.

[20] Koshkina O., Westmeier D., Lang T., Bantz C., Hahlbrock A., Würth C., Resch-Genger U., Braun U., Thiermann R., Weise C. (2016). Tuning the Surface of Nanoparticles: Impact of Poly(2-ethyl-2-oxazoline) on Protein Adsorption in Serum and Cellular Uptake. Macromol. Biosci..

[21] Dirauf M., Grune C., Weber C., Schubert U.S., Fischer D. (2020). Poly(ethylene glycol) or Poly(2-ethyl-2-oxazoline)—A Systematic Comparison of PLGA Nanoparticles from the Bottom Up. Eur. Polym. J..

[22] Holick C.T., Klein T., Mehnert C., Adermann F., Anufriev I., Streiber M., Harder L., Traeger A., Hoeppener S., Franke C. (2025). Poly(2-ethyl-2-oxazoline) (POx) as Poly(ethylene glycol) (PEG)-Lipid Substitute for Lipid Nanoparticle Formulations. Small.

[23] Kim Y., Park E.J., Kim T.W., Na D.H. (2021). Recent Progress in Drug Release Testing Methods of Biopolymeric Particulate System. Pharmaceutics.

[24] United States Pharmacopeia (2013). General Chapter <1092>: The Dissolution Procedure: Development and Validation. https://www.uspnf.com/sites/default/files/usp_pdf/EN/USPNF/gc_1092.pdf.

[25] Tang G., Zhang H., Zhao Y., Zhang Y., Li X., Yuan X. (2012). Preparation of PLGA Scaffolds with Graded Pores by Using a Gelatin-Microsphere Template as Porogen. J. Biomater. Sci. Polym. Ed..

[26] Ilyas A., Islam M., Asghar W., Menon J.U., Wadajkar A.S., Nguyen K.T., Iqbal S.M. (2013). Salt-Leaching Synthesis of Porous PLGA Nanoparticles. IEEE Trans. Nanotechnol..

[27] Huang F., Yang S., Wang H., Zhao P., Zhou B., Cheng B., Dong S., Yang J., Li B., Wang X. (2023). pH-Responsive PLGA/Gelatin Porous Microspheres Containing Paclitaxel Used for Inhibition of Cancer Cell Proliferation. J. Drug Deliv. Sci. Technol..

[28] Zhang C., Bodmeier R. (2024). Porous PLGA Microparticles Prepared with Nanosized/Micronized Sugar Particles as Porogens. Int. J. Pharm..

